# Preparation of Protein Oleogels: Effect on Structure and Functionality

**DOI:** 10.3390/foods9121745

**Published:** 2020-11-26

**Authors:** Annika Feichtinger, Elke Scholten

**Affiliations:** Physics and Physical Chemistry of Foods, Wageningen University & Research, P.O. Box 17, 6700 AA Wageningen, The Netherlands; annika.feichtinger@wur.nl

**Keywords:** oleogelation, protein, protein oleogelation, oil structuring, gel structure, gel strength

## Abstract

Among available structuring agents that have been used to provide solid properties to liquid oils, protein is a more recent candidate. Due to their nutritional value and high consumer acceptance, proteins are of special interest for the preparation of edible oleogels as an alternative for solid fats. Whereas the field of protein oleogelation is still rather new and just starts unfolding, several preparation methods have been demonstrated to be suitable for protein oleogel preparation. However, there is limited knowledge regarding the link between microstructural properties of the gels and macroscopic rheological properties, and the potential of such protein-based oleogels as a fat replacer in food products. In this review, we therefore provide an overview of various protein oleogel preparation methods and the resulting gel microstructures. Based on the different structures, we discuss how the rheological properties can be modified for the different types of protein oleogels. Finally, we consider the suitability of the different preparation methods regarding potential applications on industrial scale, and provide a short summary of the current state of knowledge regarding the behavior of protein oleogels as a fat replacer in food products.

## 1. Introduction

In recent years, the search for alternative routes to structure oil has gained increasing interest. Structured oils are referred to as oleogels, in which the continuous lipid phase is an edible oil, and the structuring agent forms a three-dimensional network. Such oleogels are used as a substitute for solid fats, which contain high amounts of saturated and trans fatty acids. As the intake of trans fatty acids and often also saturated fatty acids has been associated with health-related concerns [1,2,3,4,5], the Food and Agriculture Organization (FAO) recommends to decrease the consumption of saturated fatty acids [6]. In addition, many food products contain plant-based fats with solid properties, such as palm oil or shea butter. In addition to their high saturated fat content, a second aspect to consider is their environmental impact. The harvesting of such exotic fats has been linked to deforestation and monoculturing, which reduces biodiversity [7]. Using locally grown and more diverse plant-based liquid oils, as for example sunflower oil, would provide a potential solution [8]. Using oils is thus beneficial from a health point of view as well as a sustainability point of view.

As already shortly outlined, one possible approach is to structure liquid oils by the use of suitable structuring agents. Several types of possible alternative structuring agents have already been identified. They can be divided into low molecular weight organogelators (LMWOGs) and biopolymers. LMWOGs are often amphiphilic and self-assemble into supramolecular structures, building space-spanning networks [9]. Examples of LMWOGs that have been shown to form oleogels are monoglycerides [10,11,12,13,14,15,16,17,18], natural waxes [17,19,20,21,22,23], enzymatically synthesized wax esters [24], ceramides [25], hydroxylated fatty acids [26,27], lecithin [28,29], and oligopeptides [30]. Additionally, combinations of LMWOGs have been studied, such as fatty acids and fatty alcohols [31,32], oleic acid and sodium oleate [33], sorbitan tri-stearate, tocopherol, phytosterol, β-citosterol or ceramide in combination with lecithin [34,35,36,37,38], γ-oryzanol and β-sitosterol [39,40,41,42,43], monoglycerides and phytosterols [44], beeswax and β-carotene [45], wax and monoglycerides [46], and combinations of fully hydrogenated oil, candedilla wax and monoglycerides [47]. 

Additionally, biopolymers have been used as oil structurant, although this is less common. The use of biopolymers is more complex, as they are often hydrophilic and therefore more difficult to introduce into an oil phase. Due to its hydrophobic nature, ethylcellulose has been shown to be an effective oleogelator by direct dispersion, alone or in combination with surfactants [48,49,50,51,52,53,54,55,56,57,58,59,60,61,62,63,64,65,66]. Another biopolymer with rather hydrophobic properties used to structure oil is chitin [67,68]. Alternatively, polysaccharides can also be applied as a structuring agent with a more indirect method, such as the foam- or emulsion-templated approach. In this case, the polysaccharide should have some emulsifying properties, or other emulsifiers are used as a co-structuring agent. In this method, next to pectin [69], mostly cellulose derivatives have been used as a structurant [70,71,72,73,74,75,76,77,78,79,80,81,82].

In addition to these polysaccharides, also proteins can be used, although their potential as an oil structuring agent has received comparably little attention. Protein may be more interesting than other structuring agents that have already been investigated more extensively regarding several aspects: the nutritional value of proteins is commonly recognized, they are widely accepted by consumers, and they are label-friendly [83]. Such accepted ingredients may enhance the use of structured oils, or oleogels, in different types of food applications. Even though we know how to structure oil, it is still a technological challenge to provide structured oils that can indeed replace solid fats, as fat has several functionalities in food. Depending on the type of food, fat acts, for example, as a structure breaker, an energy absorber, a filling agent, or a lubricant. For example, in cookies, meat products or bread spreads, solid fats substantially contribute to the characteristic textural properties of the products in different ways. Therefore, for applications in food products, the structure and the physicochemical properties of the oleogels are important.

In this review, we will focus on the potential of proteins to create edible oleogels. First, we give an overview of the different preparation methods, with a specific focus on the resulting gel microstructure. Then, possibilities to modify the structure of the different oleogel-types will be discussed, and differences between the resulting rheological properties of the various gels will be compared. Subsequently, their potential applicability in food products will be evaluated, and a short summary on the current progress of application tests of oleogels and specifically protein oleogels in food products will be given. The review paper closes with an outlook on future developments and challenges.

## 2. Protein Oleogel Preparation Methods

**Figure 1:**
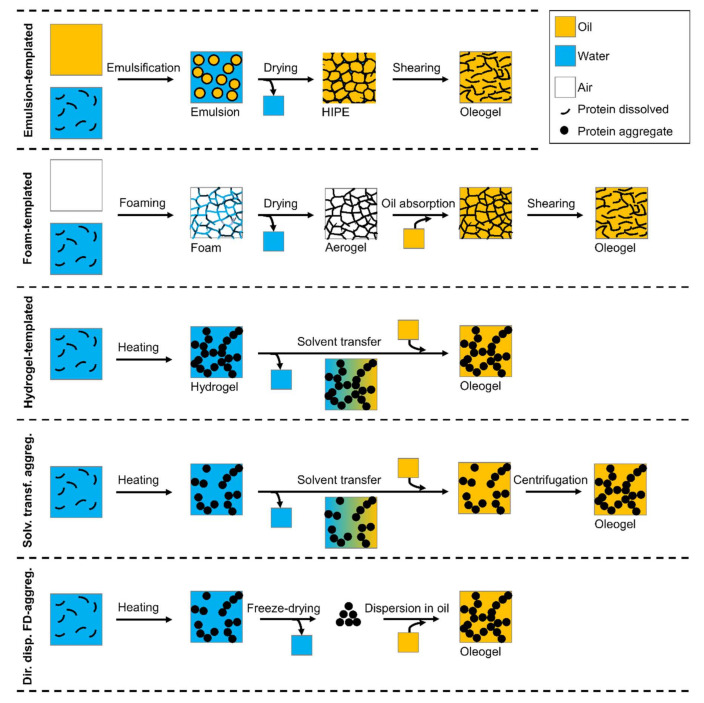
Schematic overview of the different methods for protein oleogel preparation.

As proteins are predominantly hydrophilic, it is not a straightforward exercise to introduce proteins into an oil phase, which is of hydrophobic nature. In the last years, several methods have been developed to prepare protein oleogels, sometimes also in combination with polysaccharides. Figure 1 shows a schematic overview of the different methods. Some can be regarded as indirect methods, such as the templated approaches, whereas other methods have a more direct approach.

### 2.1. Emulsion-Templated Approach

A commonly used method is the emulsion-templated approach, which was applied for the first time in 2006 by Mezzenga et al. [84] with β-lactoglobulin [84]. The general idea of this method is to first prepare an oil-in-water emulsion with proteins as an emulsifier. Polysaccharides are often added to reinforce the interface. A sufficiently stable interface is crucial for the following step, in which the water is removed by drying. This leads to the formation of a high internal phase emulsion (HIPE), consisting of a three-dimensional network of polymers filled with liquid oil. The dried emulsions are obtained by drying in an oven at elevated temperatures [85,86,87,88,89,90], by freeze-drying [85,88,89], or by drying at room temperature [84]. In these systems, the oil itself is not structured, but is held together in a network of proteins and polysaccharides. Although these dried emulsions are often referred to as oleogels, technically they are not, as the structuring agent is not the dispersed phase that creates a three-dimensional network. In such dried emulsions, the hydrophilic protein/polysaccharide network is still the continuous phase, whereas the oil is the dispersed phase. To make a real oleogel in which the oil is the continuous phase, the dried emulsion must be sheared, which breaks the continuous polymer network to obtain an oil-continuous material.

As already mentioned, a stable interface is crucial to prepare emulsions stable enough to endure the drying process. In some cases, such a stable interface was already obtained by using proteins only. Abdolmaleki et al. [88] and Alizadeh et al. [90] prepared oleogels using 2% sodium caseinate [88,90], and Tavernier et al. [86] showed that also soy protein isolate (SPI) could be used to create an emulsion stable enough to obtain oleogels at a protein concentration of 2.5%. This was explained by the formation of a relatively thick interfacial protein layer [86]. Mezzenga et al. [84] used β-lactoglobulin to create such a thick interfacial layer, but they also applied a heat treatment or added gluteraldehyde to crosslink the proteins [84]. Additionally, in the case of the more polymeric protein gelatin, stable dried emulsions and oleogels were obtained [89], although also unstable systems have been reported when using gelatin alone [85].

Even though these examples show that proteins on their own can be sufficient to create stable systems, the stability is often increased by using a combination of proteins and polysaccharides. The polysaccharides may be situated at the interface together with the protein due to attractive protein–polysaccharide interactions (co-adsorption), or reside in the continuous phase. Studied protein–polysaccharide combinations are gelatin and flaxseed gum [89], gelatin and xanthan gum [85], soy protein and k-carrageenan [86], sodium caseinate and alginate [87], and whey protein isolate and low methoxyl pectin [91]. Furthermore, oleogels have been prepared based on ternary mixtures of sodium caseinate, xanthan gum and guar gum [88]. For protein–polysaccharide combinations, the strength of the interface and the network between the oil droplets is influenced by different types of interactions, the pH-value and ionic strength of the solution. It can be commonly expected that a direct strengthening of the interface can be achieved by electrostatic interactions between the proteins and polysaccharides, often obtained at low pH, where most proteins bear a positive charge and anionic polysaccharides a negative charge. When the polysaccharides originally reside in the continuous phase (for example due to electrostatic repulsion between proteins and polysaccharides), they can be assumed to become an inherent part of the interfacial structure during drying. Upon drying, the volume fraction of the continuous phase is reduced to a large extent, which also pushes the polysaccharides towards the interfaces.

From the available studies, we see that several polysaccharides have been used to sufficiently stabilize the droplets during the drying step. As only the proteins are surface active, the concentration of proteins often determines the obtained size of the oil droplets [85,91]. The increased stability of emulsions upon polysaccharide addition is attributed to different effects, depending on the specific system. Next to co-adsorption of the polymers at the interface, also the viscosity increase of the continuous phase due to the presence of polysaccharides [89] and network formation of polysaccharides in the continuous phase [85] are assumed to contribute to emulsion stability. However, the effects of a more stable emulsion due to network formation of polysaccharides in the continuous phase seems to be a stability-enhancing factor only if these polysaccharides are not located in the continuous phase at the expense of their adsorption at the interface [86,87]. Whereas in some cases complexation seems to lead to an increased adsorption at the interface [86], in other cases less adsorption is observed, and more extensive complexation led to a larger amount of polymers forming a network in the continuous phase. This can then lead to a less stable system [87].

From the described observations in these studies, it becomes clear that the stability of the emulsions can be increased in different ways. However, it is not fully understood yet which parameters are most important, and knowledge on the effect of different interactions between proteins and polysaccharides is limited. Only interfacial tension and zeta-potential measurements have been reported. However, although these parameters are important for the stability of emulsions, they may not be the most appropriate parameters for very concentrated emulsions. During drying, the droplets are pushed together, and therefore the properties of the interface and the concentrated polysaccharide film between the oil droplets are more relevant to decrease the possibility of droplet coalescence. In this case, the thickness of the interfacial protein or polysaccharide layer becomes more important. Not the surface tension, but the viscoelastic properties, measured as the elastic and viscous modulus of the interface, should be taken into account. No studies report how much polysaccharide is located at the interface, or mention the thickness of the adsorbed layer and the viscoelastic properties of the interfacial layer. It is therefore not fully known yet which parameters are most important for emulsion stability at such high dispersed volume fractions.

### 2.2. Foam-Templated Approach

Similar to the emulsion-templated approach, also the foam-templated approach is based on freeze-drying of a previously prepared foam. After the drying step, oil is then added to the obtained dried foam-templates until saturation is reached [92,93], or the template is immersed in oil to let the dried foam absorb the oil in its voids [90,94]. As the oil is added later, the final amount of incorporated oil depends on the absorption capacity of the dried foams (aerogels). Additionally for foams, it is important that the interfaces are stable enough to prevent coalescence of the air bubbles during drying. Although stable systems have been found with proteins only (gelatin, sodium caseinate) [90,92], additional polysaccharides are often added, such as xanthan gum [92,93], hydroxypropyl methylcellulose [90], and alginate [94]. In the case of the gelatin-based foam, additional polysaccharides were not a required component to obtain a sufficiently stable foam, but the addition of xanthan considerably increased the oil sorption capacity of the resulting cryogels [92]. Sodium caseinate alone was sufficient to form a foam-templated oleogel, but addition of hydroxypropyl methylcellulose increased the gel strength [90]. After the oil is absorbed by the dried foam, a continuous protein–polysaccharide network with oil in the voids as the dispersed phase is obtained. To prepare real oleogels in which the oil is the continuous phase, the dried foam needs to be sheared to break down the polymer network. This was done in the research of Abdollahi et al. [92] and Alizadeh et al. [90] based on gelatin and sodium caseinate, respectively, resulting in oleogels with an oil binding capacity of more than 90% [90,92]. This demonstrates that also the foam-templated method is a feasible approach for protein oleogel preparation.

### 2.3. Hydrogel-Templated Approach

De Vries et al. [95] obtained oleogels solely containing oil and protein (~10–16%) based on hydrogel-templates. First, protein hydrogels were obtained by heating of whey protein isolate (WPI)-solutions. Then the water-continuous phase of the hydrogels was replaced with sunflower oil by a stepwise solvent transfer procedure, in which either acetone or tetrahydrofuran was used as intermediate solvent. Due to the slow change in solvent polarity, most of the water was removed without inducing collapse of the protein network, and only 1% of water was left in the final oleogels. Consequently, in this approach, the resulting protein oleogels consist of a three-dimensional network of dispersed proteins, which structures the continuous oil phase. Depending on the ionic strength used during formation of the hydrogels, the structure of this three-dimensional network was defined as either a fine stranded or coarse network. As the network of the hydrogels remained intact during the solvent transfer procedure, these differences in network structure were also visible for the corresponding oleogels. Whereas both hydro- and oleogels were transparent at low ionic strength (fine-stranded network), they were turbid at higher ionic strength (coarse network). The oleogels showed minimal oil leakage over several weeks [95].

### 2.4. Solvent Transfer Method with Protein Aggregates

Another method developed by De Vries et al. [96] is the preparation of oleogels based on heat-set whey protein aggregates. Protein aggregates are formed by heating of an aqueous protein solution, and similar to the previously described method, the water is replaced with oil using an intermediate solvent by centrifugation and subsequent replacement of the solvent. In the final step, a dispersion of protein aggregates in oil is obtained, and gel formation is induced by centrifugation. The small amounts of water remaining in the obtained oleogel samples (~0.8%) showed that the solvent transfer method is an efficient procedure for water removal. Oleogels with solid-like properties were obtained at a protein concentration of 3%. The particle size (*d*_4,3_) of protein aggregates in oil was with ~200 nm comparable to the particle size in water before the solvent transfer procedure [96].

### 2.5. Direct Dispersion of Freeze-Dried Aggregates

As the previous described “solvent transfer” method is rather laborious and requires large amounts of solvents, it was also attempted to obtain a protein aggregate powder by freeze-drying, and to subsequently directly disperse this freeze-dried material into oil. De Vries et al. [97] showed that it was indeed possible to obtain a freeze-dried protein powder that could be used directly to form oleogels. A crucial factor during the freeze-drying process was to limit the agglomeration of protein aggregates due to the enhanced capillary forces during drying. This could be done by (i) faster freezing of the protein aggregate solution using liquid nitrogen or (ii) drying the protein aggregates from a solvent with low polarity to reduce the capillary forces. Although not all protein agglomeration was prevented, after removal of large aggregates by centrifugation, the resulting particle size distribution was comparable to that of the aggregates obtained by the solvent transfer method [97].

Similar results were obtained by Plazzotta et al. [98], who next to freeze-drying also obtained dried whey protein aggregates by supercritical CO_2_-drying. Before the CO_2_-drying was applied, protein aggregates were first transferred from water to ethanol, which was then replaced by CO_2_. They found that freeze-drying led to considerably larger particles (*d*_4,3_ ~700 nm) compared to CO_2_-drying (*d*_4,3_ ~300 nm), indicating that CO_2_-drying was more efficient to prevent protein agglomeration. It was assumed that the slow change in polarity of the medium in which the protein aggregates were dispersed during the solvent transfer (water, ethanol, CO_2_) promoted particle–solvent interactions instead of particle–particle interactions, and therefore less agglomeration was obtained upon removal of the solvent. In their research, only the smaller particles of 300 nm obtained by CO_2_-drying were able to form a self-standing oleogel, which was related to their larger available surface area to induce particle interactions [98].

## 3. Comparison of Resulting Oleogel-Structures

**Figure 2:**
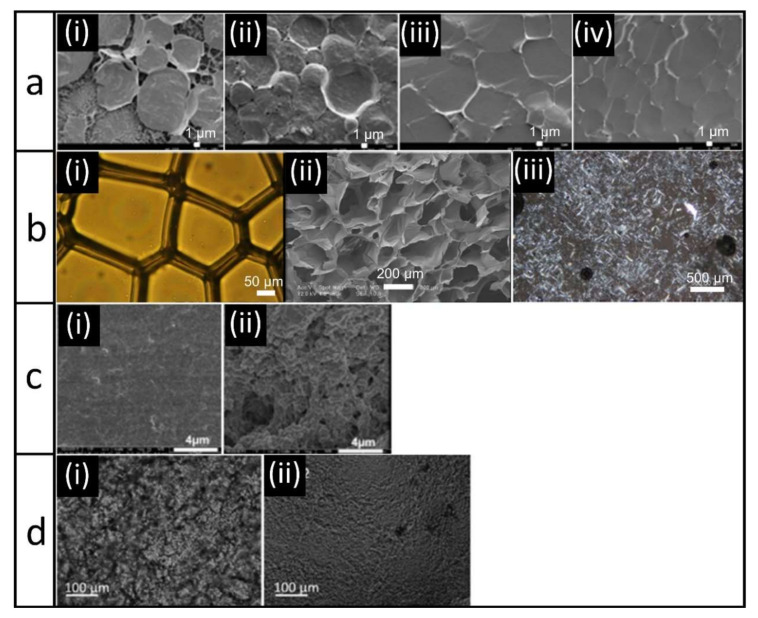
(**a**) Cryo-SEM images of emulsions at pH 5.5 (i) and pH 7 (ii) and corresponding oleogels (iii,iv) based on caseinate and alginate. Images were reproduced from Wijaya et al. [87] with permission from Elsevier; (**b**) optical image of aqueous foam (i), electron image of aerogel (ii), and polarized light microscopy image of oleogel (iii) obtained from a combination of gelatin and xanthan gum. Images were reproduced from Abdollahi et al. [92] with permission from Wiley; (**c**) Cryo-SEM images of whey protein isolate (WPI) oleogels obtained from hydrogel templates at low (i) and high (ii) ionic strength (0 or 100 mM NaCl) [95]. All images were reprinted from Langmuir 2015, 31, 13850–13859. Copyright 2015 American Chemical Society; (**d**) Optical microscopy images of an oleogel prepared by direct dispersion of freeze-dried (i) or CO_2_-dried (ii) WPI-aggregates. Images were reproduced from Plazotta et al. [98] with permission from Elsevier.

As outlined in the previous section, a variety of methods to prepare protein oleogels have been developed in the past two decades. These different methods lead to diverse structures of the resulting oleogels, and will thus influence the macroscopic properties of the oleogels. We will therefore compare the different structures obtained. Microscopic images of the gels prepared with varying approaches are given in Figure 2. 

The structure of oleogels obtained with the emulsion-templated approach remind of a honeycomb; the oil is the honey filling the combs, whereas the dried interface provides the comb structure. Figure 2a provides microscopy images taken from the research of Wijaya et al. [87]. Clearly, the originally spherical emulsion droplets are now deformed. Figure 2a, images i and ii show emulsions for which the interactions between caseinate and alginate were altered by changing the pH. At a pH close to the pI (Figure 2a-i), it can be seen that the formed complexes not only adsorbed at the interfaces, but also formed a network in the continuous phase. For repulsive interactions (Figure 2a-ii), such network formation is less visible. The resulting oleogels, i.e., dried emulsions after shearing, are shown in Figure 2a-iii,iv [87]. Without shearing, it would be expected that the droplets are deformed due to close packing but still intact. This is also visible in the images. However, after shearing, we would expect that the droplets are not stabilized anymore due to disruption of the network, and we would not expect to still see defined “oil droplets”. This shows that shearing obviously does not lead to complete disruption of the network, and that stabilized oil droplets are still present as the dispersed phase.

Similar structures are observed for foam-templated oleogels. We take examples of foams stabilized by gelatin and xanthan gum (Figure 2b) [92]. Before drying (Figure 2b-i), the air bubbles are larger and more polyhedral than the spherical oil droplets in emulsions, and thus the structures of the dried foams (Figure 2b-ii) are also more irregular. In addition, compared to the emulsion-templated approach, the foam structure is considerably more distorted by the drying step, which also affects the structure of the final oleogel (Figure 2b-iii). In this case, it seems that no “droplet-shape” structures are present in the oleogels, indicating that the structure was indeed broken enough after shearing to transfer the oil phase from the dispersed phase to the continuous phase [92]. Due to the lower magnification used for the images of the foam-templated oleogel, it is difficult to assess its structure in comparison to oleogels obtained with the emulsion-templated approach. However, it does seem to be the case that for the foam-templated approach, shearing the system led to a change in the structure, whereas for the emulsion-templated approach, the oil droplets still seemed to be entrapped within a polymer network.

Such “droplet” structures observed for the emulsion and foam-templated method are not visible in the oleogels obtained by the hydrogel-templated approach (Figure 2c). Here, the oil is held by a dispersed network of protein. The microstructure of the gels is uniform without oil accumulations or voids, which indicates that the network of the hydrogel remained undamaged. The differences in the structures shown in the two images are a result of the differences in ionic strength. At a higher ionic strength (Figure 2c-ii), the coarseness of the network increased compared to a lower ionic strength (Figure 2c-i) [95].

For protein aggregates, we see a well-defined network of proteins [96,98]. Figure 2d shows images of oleogels prepared with aggregates obtained from freeze-drying and CO_2_-drying [98]. For both methods, a continuous protein network is formed, with finer pores in case of smaller particles as obtained by CO_2_-drying (Figure 2d-ii) than for larger particles as obtained by freeze-drying (Figure 2d-i). The oil is located in the pores of the protein network [98].

## 4. Factors Affecting the Rheological Properties of Oleogels

As different preparation methods lead to different structures, also the parameters that will influence the rheological properties of the final gels vary. Here we will discuss how the gel strength, measured by the storage modulus, G’, can be adjusted for the different oleogel preparation approaches.

### 4.1. Emulsion- and Foam-Templated Approach

For oleogels prepared with the emulsion- and foam-templated approach, already the choice of protein leads to a different gel strength. When 1% SPI was used, the value of G’ was found to be 100 kPa [86], but for gelatin or sodium caseinate only, a much lower G’ of 20–27 kPa was obtained for similar protein concentrations of 1.2% and 2%, respectively [88,89,90]. These results show that differences in the protein network formation at the interfaces already influence the gel properties. Different polysaccharides do not always affect emulsion stability and gel properties in the same way.

Even though the addition of polysaccharide is expected to result in stronger gels, for oleogels based on SPI, the addition of κ-carrageenan decreased the value of G’ from 100 kPa for proteins only to around 6 kPa for a combination of SPI and κ-carrageenan. This could not be attributed to a lower number of complexes that adsorbed at the interface, as the protein load was higher in case of the complexes than for protein only. Therefore, the higher gel strength for protein only could possibly be related to a higher flexibility of the un-complexed soy protein layer, resulting in a more beneficial arrangement on the interface compared to protein complexed with polysaccharide [86]. For oleogels based on gelatin, G’-values were similar for gels prepared with or without the addition of flaxseed gum [89]. These results suggest that the addition of polysaccharides does not necessarily lead to a more favorable network formation and could even result in weaker gels. However, in many other oleogels, polysaccharides contribute to gel strength or are required to make a stable system [85,88,89,90,94]. The effect of polysaccharides on the gel strength therefore seems to depend on the specific composition used.

Next to the choice of protein and polysaccharide type, another rather straightforward strategy to influence gel properties is the variation of the polymer concentration itself. The solid-like behavior often increases with an increasing polymer concentration, specifically with an increasing protein concentration [86,87,88,90]. However, it is interesting to note that an increasing polysaccharide concentration does not always lead to an increasing gel strength. Similar to the negative effect of κ-carrageenan on the gel strength of SPI-stabilized oleogels, too much polysaccharides or too strong interactions can have a detrimental effect. For example, this was shown in the research of Qiu et al. [89], who used combinations of gelatin, tannic acid and flaxseed gum. They found that addition of flaxseed gum strengthened the network, but an excess in tannic acid had the opposite effect (oil leakage during drying) [89]. They suggested that extensive interactions between gelatin and tannic acid led to a limited flexibility of the protein, and as a consequence less optimal network formation. Abdolmaleki et al. [88] showed that for sodium caseinate-stabilized emulsions at a pH above the iso-electric point of the sodium caseinate, a too high guar gum concentration led to weaker gels. As guar gum is an anionic polysaccharide, this effect could not be ascribed to extensive attractive interactions, but an increased viscosity of the continuous phase was thought to hinder the diffusion of caseinate to the interface during emulsification [88].

Another factor influencing gel strength seems to be the position of the polysaccharides already before the drying step. This becomes clear from oleogels prepared with sodium caseinate (1.2%) and alginate (0.1%). At pH-values close to the isoelectric point of caseinate, at pH 5.5, polymers were not only present at the interface, but network formation was also observed in the continuous phase. Consequently, rather low values for G’ of 35 kPa were observed. At higher pH-values of 7, when repulsive interactions were present, less complexation occurred, and no network formation was observed in the continuous phase. In this case, higher values for G’ of ~70 kPa were obtained. This was explained by a more flexible and denser interface structure [87].

Next to the type, concentration and position of proteins and polysaccharides and their interactions, also the drying process can influence the resulting gel properties. In general, oven-drying would be expected to lead to softer oleogels, due to enhanced structure damage due to the stresses resulting from capillary forces. This was indeed the case in the research of Qiu et al. [89], whereas the opposite result was reported in the study of Abdolmaleki et al. [88]. No other literature is available on the effect of drying techniques. From current research, it is therefore not yet clear how different drying techniques influence the stability of the emulsions, and the properties of the resulting oleogels.

### 4.2. Hydrogel-Templated Approach

For oleogels based on WPI-hydrogels, the gel properties were determined by the protein content and the network structure, i.e., a coarse or a fine-stranded network. A higher protein content or coarser gel network resulted in an increased gel stiffness, measured as Young’s modulus. Fine stranded oleogels with 15% protein had a modulus of around 600 kPa, whereas coarse gels gave moduli of more than 10,000 kPa. These values were much higher than the ones found for the corresponding hydrogels. For example, for fine-stranded hydrogels (protein content 16%), the modulus was around 100 kPa, whereas the coarse hydrogels showed values of 7000 kPa. Oleogels were thus stiffer than the corresponding hydrogels. The 70 times higher gel stiffness is explained by the increased interaction between the proteins in oil due to a lower solvent quality. The stronger interactions also increased the brittleness of the oleogels in comparison with the hydrogels [95].

### 4.3. Network Formation of Protein Aggregates in Oil

For both the solvent transfer method of heat-set protein aggregates and direct dispersion of protein aggregates in oil, network formation of the protein aggregates occurs after introduction into the oil phase. In case of the solvent transfer method, the resulting protein concentration is mainly determined by the centrifugation step, whereas for direct dispersion of freeze-dried protein aggregates, the protein concentration can be easily adjusted. To tune the final gel properties, the interactions between particles can be modified, which have been shown to depend on different parameters.

The first important parameter that influences the gel strength is the size of the protein aggregates. Plazzotta et al. [98] obtained different sizes using freeze-drying (*d*_4,3_ ~700 nm), or supercritical CO_2_-drying (*d*_4,3_ ~300 nm). For samples prepared with the smaller particles, G’ was found to be 31 kPa, which was much higher than the value of 1.1 kPa found for the larger particles (*d*_4,3_ ~700 nm) [98]. Larger particles have a lower surface area, and therefore less particle interactions are obtained. This influence of particle size was also observed by De Vries et al. [97]. They compared freeze-dried aggregates (*d*_3,2_ ~220 nm) with aggregates obtained by the solvent transfer method (*d*_3,2_ ~140 nm). At the concentrations they used, a G’ of ~4 kPa was found for the small aggregates, whereas the samples with the larger freeze-dried protein aggregates gave a G’-value of ~3 kPa. Plazzotta et al. [98] showed with FTIR measurements, that interactions are indeed increased for smaller particle sizes, which were attributed to hydrogen bond formation [98]. These results also demonstrate that H-bonding plays a crucial role in the development of a protein network. 

The network formation of protein oleogels is mostly based on attractive interactions between the hydrophilic particles in the hydrophobic oil phase [96,99]. As these attractive interactions are of hydrophilic nature, they can be altered by addition of small amounts of water. De Vries et al. [99] showed that water-mediated interactions increase G’-values to a large extent. The highest increase in gel strength was obtained for water addition of 0.5 g water/g protein. For example, at a protein content of 8%, the value of G’ increased by two orders of magnitude from 0.5 kPa to 40 kPa. Next to the formation of hydrogen bonds, this is most likely a result of the formation of capillary bridges. An additional heat treatment visibly increased gel strength further. CLSM images showed enhanced clustering and contraction of the protein network, which indicated that heating led to rearrangements of the network structure due to increased mobility, resulting in a denser network with higher strength [99].

Instead of changing the attractive interactions, also the strength of the repulsive interactions can be altered to change the gel strength. These repulsive interactions between protein aggregates and the continuous oil phase can be influenced by changing the polarity of the oil. Use of more polar oils led to weaker network formation, as indicated by a decreasing value of G’ when exchanging medium chain triglyceride oil by sunflower oil or olive oil (increasing polarity) [100]. This is explained by enhanced interactions between the mostly hydrophilic protein particles and the more polar oil phase, and therefore weaker interactions between the protein aggregates.

## 5. Rheological Properties of Oleogels in Relation to Gel Structure

To draw conclusions on how efficient the different oleogel preparation methods are regarding high gel strengths in combination with low amounts of protein, i.e., structurant, values of the storage modulus G’ are summarized in Table 1. It has to be noted that some variations regarding equipment, the specific measurement geometry, and measurement methods between different studies are present. Whereas a comparison of precise values is therefore only possible for presented values obtained from the same study, the results of different studies can still be compared, but based on the differences in orders of magnitude. 

For the emulsion- and foam-templated oleogels, a rather broad range of G’-values was obtained in different studies, which for the chosen examples shown in Table 1 ranged between 0.5 and 400 kPa. As discussed previously, the differences in gel strength for oleogels prepared with varying proteins indicate that the choice of the protein may already be important. The effect of polysaccharide addition depends on the specific system, and can next to an increased gel strength [88,90] also result in a comparable [89] or decreased [86] gel strength.

For oleogels structured by a three-dimensional network of protein aggregates, as obtained either by the solvent transfer method or direct addition of dried protein powder, G’-values were lower even at higher protein concentrations. For example, in the solvent transfer method of protein aggregates, a whey protein concentration of 2% resulted in a G’-value of 0.1–0.2 Pa, 8% in 700 Pa [99], and 10% in a G’-value of around 4000 Pa [100]. To reach a comparable value for G’ as for the templated methods, protein concentrations of 15% were needed, which increased the G’-value to 300 kPa (oleogel obtained by direct dispersion of protein particles) [98]. However, these low G’-values for low protein concentrations could be easily increased by addition of small amounts of water. For example, for an oleogel with 8% protein, upon water addition, a G’-value of around 40 kPa was obtained [99]. Compared to the templated methods, it therefore seems to be easier to adjust the gel strength of oleogels based on network formation of protein aggregates. Interactions can still be changed, and protein concentration itself has a large effect.

The fact that interactions in the emulsion- and foam-templated oleogels do not seem to have a clear effect on the gel strength, whereas this is more obvious for the oleogels prepared with protein aggregates, can be attributed to the difference in the network structure. For the emulsion- and foam-templated approach, the added polymers will arrange in evenly distributed and thin layers throughout the three-dimensional space. After drying, this continuous polymer phase will then enclose large pools of the liquid oil phase. The interactions between the ingredients do not seem to be the determining factor here, but other factors that are not yet understood. They seem to be linked more to the interfacial properties or the highly concentrated network in the continuous phase. On the contrary, when using protein aggregates, the protein is actually the dispersed phase. In this case, a clear trend is observed for protein concentration and the interactions between the proteins.

Oleogels prepared by the hydrogel-templated approach provide the largest gel strength. The gels were characterized by compression tests. As Young’s modulus is related to storage modulus as E = 3G for incompressible material (Poisson ratio of 0.5), we report recalculated values in Table 1 for a more direct comparison. We see that gels prepared with this method show much higher gel strength. For example, for hydrogel templates with a protein content of 15% and coarse network structure, a Young’s modulus of more than 10,000 kPa is obtained [95]. These high gel strengths are a result of covalent interactions (disulfide bridges) obtained when preparing the hydrogels, as initial attractive hydrophobic interactions between proteins in water become the repulsive interactions when transferred to oil. The properties of the oleogels can therefore be adjusted by altering the interactions and the network structure in the preceding hydrogels, but cannot be adjusted anymore after the oleogels are obtained. As opposed to the spreadable gels prepared by the other methods, these oleogels are characterized by a fracture behavior and no yielding is observed.

In most studies, mainly gel strength (storage modulus) is measured as a representative for the properties of the oleogels. However, for food applications, not only the gel strength, but also other rheological and physicochemical properties, as yield value, structure recovery, and shear resistance are important. Especially, structure recovery is relevant when oleogels are used on an industrial scale, where it needs to be pumped through pipes. When a material can easily recover its structure, it will be less susceptible to changes occurring during high shear conditions. In some studies, rheological measurements have been included to determine structure recovery, and the results show that these oleogels showed partial structure recovery upon shearing [85,86,88,89,92,100].

Only for oleogels prepared with the emulsion- and foam-templated approach, extensive shear and mechanical stress can lead to oil leakage as a result of a damage of the honeycomb-structure. Once a critical strain is reached, this structure may not be able to recover. This is not the case for oleogels structured by a network of protein aggregates, where the oil is the continuous phase, and the structure recovers due to strong interactions between the dispersed protein aggregates. However, this does not apply for oleogels obtained by the hydrogel-templated method; these gels will fracture upon deformation.

## 6. Oleogels for Food Applications

In the food industry, the exchange of solid fats in foods by oils has been subject of research for many years. Protein oleogels could be a potential solution. As the original composition of the oil remains unchanged, and protein is used as a structurant, these protein oleogels would be a label-friendly ingredient with high consumer acceptance. As outlined in this review, a variety of methods to prepare protein oleogels have been developed in the past two decades, resulting in oleogels with different structural and rheological properties. Next to these macroscopic gel properties, it is also important that the preparation method can be used on an industrial scale. The necessary amount of protein to obtain a specific gel strength and the use of other resources are of relevance. Taking these various aspects into account, different approaches are interesting due to different aspects.

### 6.1. Suitability of the Different Preparation Methods

For the emulsion- and foam-templated approach, the necessary amounts of gelator molecules are low (<3%). The preparation of oleogels requires emulsification, freeze-drying, and shearing, but besides the structuring agents themselves, no further materials, such as solvents, are needed. However, next to protein, mostly also polysaccharides are necessary to obtain a stable gel. Only in few studies, protein oleogels with solid-like properties could be obtained based on SPI [86], gelatin [89], or caseinate [88,90] alone.

For the hydrogel-templated approach, the addition of polysaccharides to obtain stable gels is not required. Additionally, the gel strengths are much higher, but more protein is required to obtain a stable hydrogel as the starting material. Regarding the preparation procedure, the downside of this approach is the use of large amounts of solvent, as several washing steps are necessary. This makes the approach laborious and not very environmentally friendly. The same problem is observed for the “solvent transfer” approach for the protein aggregates, although the preparation time is less as for the hydrogels.

When using the protein aggregates, it has already been shown that the application of solvent can be prevented. Instead, an aqueous suspension of protein aggregates can be dried to a protein powder and directly added to oil. Here the drying step is a critical choice, as it will determine the final particle size of the obtained protein powder. During freeze-drying, agglomeration of the protein aggregates may still take place. In the case when too much agglomeration occurs, a higher amount of protein is needed to obtain gels with a certain gel strength. It has already been shown that if the drying step is done with CO_2_-drying, smaller particles can be obtained. Further optimizations in the drying processes could be made to obtain a desired finer powder with smaller particles for more efficient network formation.

Another major benefit of preparing oleogels based on heat-set protein aggregates is the expected universal applicability with different types of globular proteins. Here, also the possibility to develop sustainable and vegan products is a promising perspective. For the emulsion- and foam-templated approach, on the contrary, the use of protein alone seems to be limited to specific protein sources, and might even be sensitive to differences between protein isolates obtained from different producers.

### 6.2. Potential as Solid Fat Replacer in Foods

Up to now, there are no extensive studies on the application of protein oleogels in food products available, but mainly oleogels based on polysaccharides have been tested in several application studies. Oleogels based on ethylcellulose have been used in meat applications like sausages [49,50,51,52,53,54,55], but also in bread [58], ice cream [101], chocolate [102], and as antibacterial edible packaging [56]. Oleogels obtained by the emulsion-templated approach structured with methylcellulose [71] and pectin [69] have been tested in sponge cakes. Foam-templated oleogels prepared with methylcellulose have been tested in meat patties [77] as well as in bakery products [76] and spreads [80,81]. In general, these studies show that replacement of solid fats by oleogels can result in products with textural and sensory properties comparable to those of the reference products containing solid fats. However, this was mostly only the case for partial solid fat replacement. Complete fat replacement therefore still seems challenging. To replace all fat, further in-depth studies should be performed to understand the optimal physicochemical properties of the oleogels for different type of food applications.

For oleogels prepared based on protein, a foam-templated oleogel prepared by using pea protein concentrate and xanthan gum was tested in cake [93], but no further studies seem to be available. Especially for oleogels prepared by the use of protein alone, no elaborate tests for its use in food products have been conducted yet. Nevertheless, a small study on cookies as well as sausages with WPI-oleogels was performed [103]. Simple replacement of solid fat by oil showed a clear negative effect in the structure of the resulting products, for cookies as well as the sausages. When the solid fat was replaced by protein oleogel, the obtained products were largely comparable in appearance and texture profiles to the standard products. For the cookies, replacement of solid fat by protein oleogel resulted in a decreased hardness and therefore also decreased crunchiness. In the case of the sausages, the fracture properties were comparable for the reference product and the oleogel-containing product, and no large differences in sensory perception were found [103]. These tests are a first indication that protein oleogels can provide a suitable replacement for solid fats in foods, but clearly in-depth studies on the functionality of such oleogels in different categories of foods are still missing. Next to the effects of fat replacement by protein oleogels on functional and sensorial properties, also the effects of oxidation reactions occurring in the gels, which involve proteins as well as lipids, need to be studied in the context of their use in food products. These oxidation reactions, which especially occur when water is present, may have a detrimental impact on the nutritional value of the gels [104,105].

## 7. Concluding Remarks and Outlook

From research on protein oleogelation performed so far, future applications of protein oleogels in food products seem to be a promising perspective. Even though the field of liquid oil structuring based on proteins is rather recent and just starts evolving, a number of different methods that can be used to obtain protein oleogels have already been developed. Although the knowledge on methodologies for oleogel preparation exists and oleogels with a widespread range of rheological properties have been obtained, an in-depth understanding of the link between gel network formation on colloidal scale and properties on macroscopic scale is still missing. Such knowledge is required to design oleogels suitable for different types of applications. As one oleogel type is not expected to meet the necessary requirements for fat replacement in different products, design of different oleogel structures and properties may be needed. In addition to gel strength, also other properties, such as structure recovery, yield stress, relaxation phenomena, and behavior under different processing conditions are important. In addition, also aspects such as temperature sensitivity and lipid oxidation are relevant. For the emulsion- and foam-templated approach, such properties can be controlled by knowledge on the specific role of the interfacial properties and polymer network formation in the continuous phase. In the case of the solvent transfer methods of protein aggregates and the direct dispersion of protein aggregates in oil, more knowledge is required on how specific particle properties determine the network formation and how these interactions can be modified to adjust the resulting gel properties. As in these oleogels, the liquid oil phase is indeed structured by a network of interacting protein aggregates, a wide range of possibilities to adjust the different rheological properties is available by modifications of the interactions between the protein aggregates themselves. This also provides a greater flexibility regarding the use of different protein sources. For final applications on an industrial scale, a challenge still to be solved is to find ways to circumvent the use of solvents, by further optimization of drying processes to obtain powders suitable for direct dispersion in oil. Furthermore, to be able to draw conclusions on the applicability of protein oleogels in foods, research on the effects of protein oleogel incorporation on textural and sensory properties in different types of food products still needs to be performed. However, with the current pressure on the food industry to reformulate their products with respect to health and sustainability, we are convinced that protein oleogels and other alternative methods to structure oil will remain a subject of great interest.

## Figures and Tables

**Table 1:** Examples of G’-values of protein oleogels prepared by different methods, measured by oscillatory rheology.

Approach	System	Concentration (%)	G’ (kPa)
Emulsion-templates	Gelatin (GLT) Flaxseed gum (FG) [89]	**Freeze-dried**	
		1.2% GLT	20
		1.2% GLT, 0.6 FG	20
		**Oven-dried**	
		1.2% GLT, 0.6 FG	0.5
	Gelatin (GLT) Xanthan gum (XG) [85]	**Freeze-dried**	
		0.6–1.6% GLT, 0.6–1.5% XG ^1^	20
	Soy protein isolate (SPI) κ-carrageenan (κ-car.) [86]	**Oven-dried**	
		1% SPI	100
		2.5% SPI	400
		1% SPI, 0.067% κ-car.	6
		2.5% SPI, 0.067% κ-car.	200
	Sodium caseinate (CN) Alginate (ALG) [87]	**Oven-dried**	
		1.2% CN, 0.1% ALG (pH7)	70
		1.2% CN, 0.1% ALG (conjugate)	75
	Sodium caseinate (CN) Xanthan gum (XG) Guar gum (GG) [88]	**Oven-dried**	
		2% CN	27
		2% CN, 0.5% XG, 0.5% GG	270
		**Freeze-dried**	
		2% CN	25
		2% CN, 0.5% XG, 0.5% GG	150
Foam-templates	Gelatin (GLT) Xanthan gum (XG) [92]	**Freeze-dried**	
		3% GLT, 0.2% XG	30–100
	Sodium caseinate (CN) Hydroxypropyl methylcellulose (HPMC) [90]	**Freeze-dried**	
		2% CN	25
		2% CN, 2% HPMC	235
Solvent transfer of heat-set aggregates	Whey protein isolate (WPI)	2%	0.0001–0.0002 [99]
		2%, 0.5 g water/g protein	0.2 [99]
		8%	0.7 [99]
		8%, 0.5 g water/g protein	40 [99]
		10%	4 [100]
Direct dispersion of dried particles	WPI	**Freeze-dried**	
		10%	3 [97]
		15%	1 [98]
		**CO_2_-dried**	
		15%	300 [98]
Hydrogel-templates ^2^	WPI [95]	10%, 50 mM NaCl (fine-stranded)	~350
		16%, 50 mM NaCl (fine-stranded)	~2500
		15%, 200 mM (NaCl)	~3500

^1^ exact concentration at which the oleogel was prepared could not be found. ^2^ values recalculated from Young’s modulus as G’ = E/3.
